# Redox-Sensitive Mapping of a Mouse Tumor Model Using Sparse Projection Sampling of Electron Paramagnetic Resonance

**DOI:** 10.1089/ars.2021.0003

**Published:** 2022-01-17

**Authors:** Kota Kimura, Nami Iguchi, Hitomi Nakano, Hironobu Yasui, Shingo Matsumoto, Osamu Inanami, Hiroshi Hirata

**Affiliations:** ^1^ Division of Bioengineering and Bioinformatics, Graduate School of Information Science and Technology, Hokkaido University, Sapporo, Japan.; ^2^ Division of Bioengineering and Bioinformatics, Faculty of Information Science and Technology, Hokkaido University, Sapporo, Japan.; ^3^ Laboratory of Radiation Biology, Faculty of Veterinary Medicine, Hokkaido University, Sapporo, Japan.

**Keywords:** redox-sensitive mapping, electron paramagnetic resonance, nitroxyl radical, compressed sensing, mouse xenograft model

## Abstract

***Aims:*** This work aimed to establish an accelerated imaging system for redox-sensitive mapping in a mouse tumor model using electron paramagnetic resonance (EPR) and nitroxyl radicals.

***Results:*** Sparse sampling of EPR spectral projections was demonstrated for a solution phantom. The reconstructed three-dimensional (3D) images with filtered back-projection (FBP) and compressed sensing image reconstruction were quantitatively assessed for the solution phantom. Mouse xenograft models of a human-derived pancreatic ductal adenocarcinoma cell line, MIA PaCa-2, were also measured for redox-sensitive mapping with the sparse sampling technique.

***Innovation:*** A short-lifetime redox-sensitive nitroxyl radical (^15^N-labeled perdeuterated Tempone) could be measured to map the decay rates of the EPR signals for the mouse xenograft models. Acceleration of 3D EPR image acquisition broadened the choices of nitroxyl radical probes with various redox sensitivities to biological environments.

***Conclusion:*** Sparse sampling of EPR spectral projections accelerated image acquisition in the 3D redox-sensitive mapping of mouse tumor-bearing legs fourfold compared with conventional image acquisition with FBP. *Antioxid. Redox Signal.* 36, 57–69.

**Figure:**
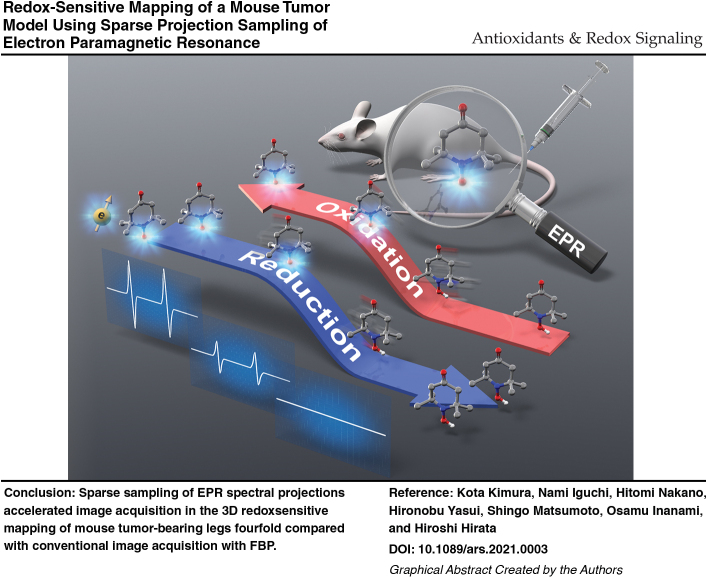
(*Color images are available online*).

## Introduction

A reduction–oxidation balance called the redox state is a critical part of homeostasis in a living organism (52). Dysfunction of redox homeostasis leads to many diseases; an altered redox state can be seen as evidence of a disease's pathological status (15). Therefore, the biomedical community desires noninvasive techniques for imaging the redox state in biological tissues (25). To date, several imaging modalities have been developed for redox-sensitive mapping in animal models of disease, including magnetic resonance imaging (MRI) (19), dynamic nuclear polarization (DNP) MRI (50), fluorescence imaging (7), photoacoustic imaging (13), and electron paramagnetic resonance (EPR) spectroscopy and imaging (9, 48). Redox-sensitive imaging techniques use exogenous redox-sensitive molecular probes, such as nitroxyl radicals for MRI, DNP-MRI, and EPR (8, 21, 40); fluorescent probes and protein for fluorescence imaging (11, 24); and fluorescent probes for photoacoustic imaging (56).

**Innovation**

Electron paramagnetic resonance (EPR) using exogenously injected nitroxyl radicals can visualize redox-sensitive maps of mouse tumor models under a time constraint. The present work applied compressed sensing image reconstruction and the sparse spectral sampling strategy to three-dimensional redox-sensitive mapping in mouse tumor models using a short-lifetime nitroxyl radical. This progress in EPR imaging accelerates spectral acquisition for redox-sensitive nitroxyl radicals from tumors and other disease models. The accelerated EPR image acquisition broadens the choices of nitroxyl radical probes with various redox sensitivities and different degrees of lipophilicity.

**FIG. 1.**
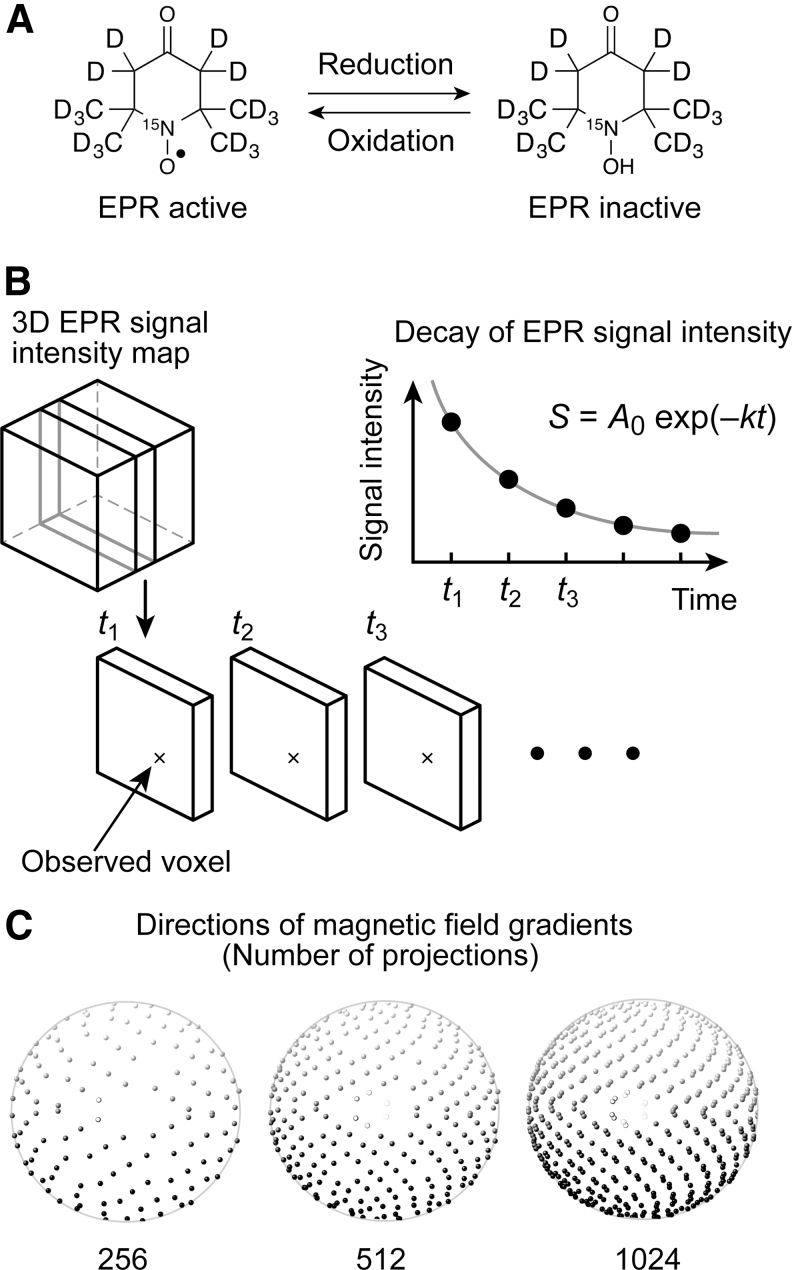
**Concept of redox-sensitive mapping with nitroxyl radicals and EPR. (A)** Structure of ^15^N-labeled perdeuterated Tempone (^15^N-PDT) and its “EPR inactive” hydroxylamine form, **(B)** concept of decay-rate mapping, and **(C)** examples of directions for EPR spectral projections with the golden mean sampling strategy. For exponential decay, *S* represents the EPR signal intensity as a function of time *t* with an initial signal intensity *A*_0_ and decay rate *k*. ^15^N-PDT, 4-oxo-2,2,6,6-tetramethylpiperidine-d_16_; 1-^15^N-1-oxyl; EPR, electron paramagnetic resonance.

EPR is the most sensitive method for the detection of nitroxyl radicals that have a single unpaired electron. Therefore, EPR imaging is used extensively as a redox-sensitive mapping technique (12). While the reduction reaction of nitroxyl radicals leads to EPR-silent hydroxylamines, the oxidation reaction of hydroxylamines leads to EPR-active nitroxyl radicals (Fig. 1A). Consequently, the redox state alters the decay rate of the EPR signals for nitroxyl radicals. EPR imaging and other imaging modalities of exogenously injected nitroxyl radicals reveal a shift in the redox state in mice with malignant tumors as a difference in signal decay (3, 18, 28, 30, 35, 44, 53). EPR-based redox-sensitive mapping is potentially applicable to disease-model animal studies; for example, for assessment of the redox state in the tumor microenvironment (27) and for monitoring the pharmacological response of the redox state in tumors involving oxidative stress, reactive oxygen species (ROS), and antioxidants (30, 36). Both applications might eventually lead to a prognosis for intervention in tumors using redox-sensitive anticancer drugs (4, 17, 31, 32). However, EPR-based redox-sensitive mapping is not limited to preclinical cancer studies; it could also be used in animal studies of other diseases involving redox homeostasis dysfunction.

In practice, a redox-sensitive map can be obtained from the temporal change in the spatial distribution of the EPR signal intensity for exogenously injected nitroxyl radicals in biological tissue. EPR image acquisition should be fast enough to follow the decay of a nitroxyl radical signal in a subject animal (Fig. 1B). A longer acquisition time for each three-dimensional (3D) image is a significant obstacle to 3D redox-sensitive mapping because 3D EPR imaging with a high spatial resolution requires many spectral projections (29). Thus, nitroxyl radicals with a short lifetime in a living organism, such as 4-oxo-2,2,6,6-tetramethylpiperidine-*N*-oxyl (4-oxo-TEMPO, also called Tempone) radical, have not been used in EPR-based redox-sensitive mapping to date.

The product of the number of spectral projections and the time required to acquire a single spectrum determines the acquisition time of a 3D EPR image. Therefore, two approaches have been used to accelerate EPR spectral acquisition. The first approach is to reduce the duration of magnetic field scanning in continuous-wave (CW) EPR detection (1, 43). A duration of field scanning of 3.8 ms has been reported in state-of-the-art CW-EPR imaging (39). Moreover, rapid-scan EPR detection was developed and has been demonstrated to be a faster means of image acquisition (47). However, such state-of-the-art hardware for EPR spectroscopy and imaging is available at only a few instrument-development laboratories.

The second approach is to reduce the number of spectral projections acquired (Fig. 1C). Usually, over-reduction of the number of spectral projections degrades the spatial resolution and the signal-to-noise ratio (SNR) of reconstructed images. Even though the number of spectral projections is reduced, relatively uniform angular distributions of spectral projections help prevent image-quality degradation with fewer spectral projections. For example, the 3D golden mean (GM) sampling method can cover a sphere with relatively uniform angular distributions (5). In contrast, compressed sensing (CS), also called sparse sampling, was reported in MRI in 2007 (33). CS is a signal processing technique for solving underdetermined linear systems using the sparsity of a signal. CS has changed the data acquisition and reconstruction strategy in MRI. Johnson *et al.* demonstrated 3D EPR imaging acceleration regarding an isolated rat heart and mouse gastrointestinal tract from a smaller number of spectral projections using the CS strategy (22). Such CS-based mapping was performed using stable particulate radicals such as activated charcoal and lithium octa-*n*-butoxynaphthalocyanine (LiNc-BuO). In contrast to stable radicals, rapidly decaying EPR signals limit the number of spectral projections obtained.

This article describes sparse projection sampling to accelerate image acquisition in EPR-based redox-sensitive mapping of a mouse tumor model. To tackle this challenge, we developed an accelerated EPR-based redox-sensitive mapping system by deploying fast-scan CW-EPR acquisition at 750 MHz (41, 43), sparse modeling (22), the 3D GMs sampling strategy (5), fast iterative shrinkage-thresholding algorithm (FISTA) (2), and an improved multiple-image-reconstruction protocol. Moreover, to demonstrate the imaging system's advances, we performed 3D mapping of a nitroxyl radical solution sample and redox-sensitive mapping in a mouse tumor model.

## Results

### EPR imaging of a solution phantom

**FIG. 2.**
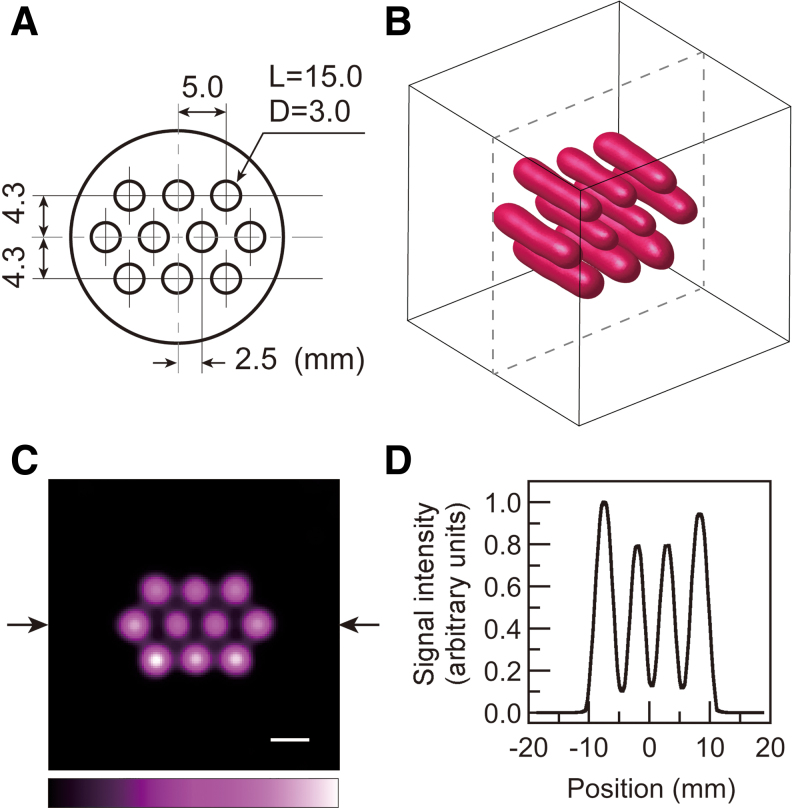
**EPR imaging of the multiple pillar solution phantom. (A)** Cross-section of the multiple pillar phantom (*L* is the length of a hole, *D* is the diameter of a hole; values are given in millimeters), **(B)** surface-rendered image of ^15^N-PDT, **(C)** slice-selective signal intensity map at the center of the visualized space **(B)**, and **(D)** signal intensity profile between the two *arrows* in the intensity map **(C)**. The matrix size of the 3D image is 96 × 96 × 96, and its FOV is 28.1 mm × 28.1 mm × 28.1 mm. For the slice-selective map, the image matrix and the FOV are 128 × 128 and 37.5 mm × 37.5 mm, respectively. The *white* scale bar on the images corresponds to 5 mm. 3D, three dimensional; FOV, field of view. Color images are available online.

A solution sample of a nitroxyl radical, ^15^N-labeled perdeuterated Tempone (^15^N-PDT), was first used in EPR imaging to test our data acquisition and image reconstruction. ^15^N-PDT has narrower EPR absorption peaks due to deuterium substitution. It also provides higher EPR signal intensity due to the hyperfine structure of the EPR spectrum for ^15^N-labeled nitroxyl radicals, which have two-line absorption peaks. Moreover, since this probe has a short lifetime in living organisms, it is a useful model for demonstrating accelerated redox-sensitive mapping in mouse tumor models. These benefits led us to choose ^15^N-PDT for our study. The solution sample, called the multiple pillar phantom, had 10 holes filled with radical solution (Fig. 2A). The end of each hole was sealed with a piece of adhesive tape. A photograph of the multiple pillar phantom is given in the Supplementary Data (Supplementary Fig. S1). The surface-rendered image of the EPR signal for ^15^N-PDT (Fig. 2B) was reconstructed by the CS approach with 2048 projections. For the surface-rendered image, the field of view (FOV) and the image matrix were 28.1 mm × 28.1 mm × 28.1 mm and 96 × 96 × 96, respectively. The shape of the ^15^N-PDT solution in the holes is seen. A slice image (FOV 37.5 mm × 37.5 mm, image matrix 128 × 128) (Fig. 2C) was obtained at the center of the 3D visualized space (dashed lines in the 3D image of Fig. 2B). The one-dimensional (1D) signal intensity profile (Fig. 2D) between the two arrows was obtained from the slice-selective signal intensity map (Fig. 2C). The lower and outer solution pillars showed slightly higher signal intensities than other solution pillars. From the 1D signal profile, the full-width at half-maximum (FWHM) of the Gaussian point spread function was estimated to be 2.5 mm. This FWHM corresponds to the spatial resolution of this image. The EPR imaging results (Fig. 2) proved that our spectral data acquisition and CS image reconstruction worked adequately.

**FIG. 3.**
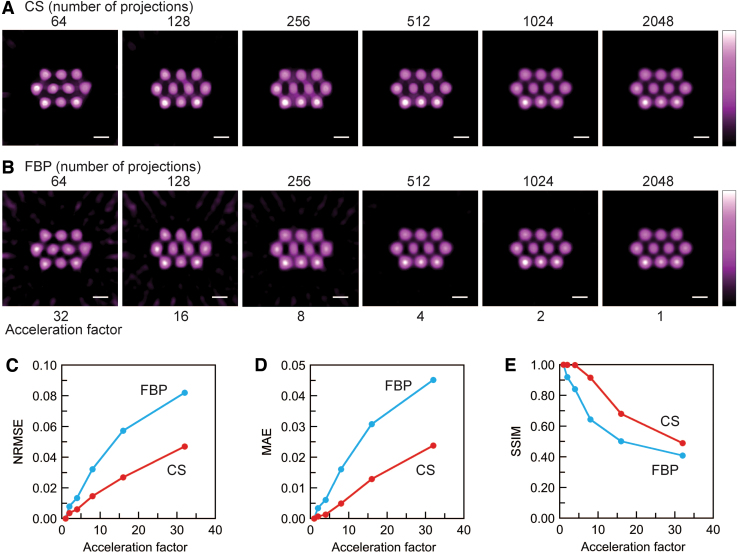
**Comparison of reconstructed images of the multiple pillar phantom with FBP and CS approaches.** Reconstructed slice-selective images with **(A)** CS and **(B)** FBP, **(C)** NRMSE, **(D)** MAE, and **(E)** SSIM. In **(C**–**E)**, the reconstructed images with 2048 spectral projections were defined as the reference images for the corresponding image-reconstruction approaches. The acceleration factor was defined as the ratio of the number of acquired spectral projections to the reference number of acquired spectral projections (2048 in this work). The *white* scale bar on the images corresponds to 5 mm. CS, compressed sensing; FBP, filtered back-projection; MAE, mean absolute error; NRMSE, normalized root mean square error; SSIM, structural similarity. Color images are available online.

Slice-selective signal intensity maps (Fig. 3A, B) were generated from CS and conventional filtered back-projection (FBP)-based 3D images with different spectral projections. Data sets of 64, 128, 256, 512, and 1024 spectral projections were retrospectively generated from the data set of 2048 measured spectral projections. The slice-selective maps are positioned at the center of the 3D space (Fig. 2C). For both approaches, an artifact in the image background was visible when a smaller number of projections were used. However, background noise was well suppressed in CS-based images compared with FBP-based images because soft-thresholding and total variation (TV)-denoising were applied in CS image reconstruction. The qualities of CS- and FBP-based images were quantitatively assessed with normalized root mean square error (NRMSE), mean absolute error (MAE), and structural similarity (SSIM) (Fig. 3C–E) (51). The CS- and FBP-based 3D intensity maps with 2048 spectral projections were used as reference images for measuring the image quality with the FBP and CS approaches. NRMSE and MAE are negative indicators, and SSIM is a positive indicator. With respect to all three indicators, CS-based images were better than FBP-based images (Fig. 3C–E). The acceleration factor is the ratio of the number of projections to 2048 (the reference in this investigation). Our sparse projection sampling and CS image reconstruction accelerated the 3D EPR image acquisition for the multiple pillar phantom by a factor of four, if we consider that the three indicators had equivalent values.

### Comparison of CS- and FBP-based images for a mouse tumor-bearing leg

We compared the 3D images of a mouse tumor-bearing leg by CS and FBP reconstruction with various numbers of EPR spectral projections to verify the image acquisition with the sparse sampling strategy for mouse tumor models. Before this imaging experiment, the time courses of the EPR signal intensity of intravenously injected ^15^N-PDT were preliminary recorded from the overall mouse tumor-bearing legs. EPR signals of ^15^N-PDT were measured from the mouse tumor-bearing legs when the tumor volume reached ∼900–1000 mm^3^. The tumor growth curve of mouse xenograft models of the human-derived pancreatic ductal adenocarcinoma cell line MIA PaCa-2 is given in the Supplementary Data (Supplementary Fig. S2). A representative EPR spectrum and time course of the EPR signal intensities are given in the Supplementary Data (Supplementary Figs. S3 and S4). These data were measured from an overall tumor-bearing leg after intravenous injection of ^15^N-PDT *via* a tail vein. The mean lifetime of the EPR signal from the tumor-bearing legs was 1.4 min for three individual measurements, corresponding to a decay rate of 0.71 min^−1^. Based on this preliminary result, we set the starting time of sequential EPR image acquisition for redox-sensitive mapping (30 s after the intravenous injection) and the number of spectral projections for a single-image scan (128 or 256 spectral projections).

**FIG. 4.**
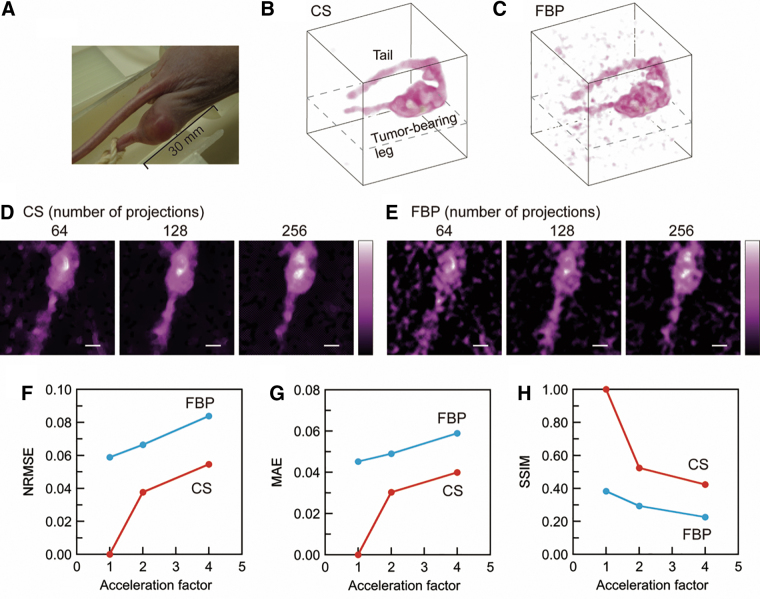
**Comparison of CS- and FBP-based reconstruction for a mouse tumor-bearing leg. (A)** Photograph of the mouse tumor-bearing leg, volume-rendered images of the mouse tumor-bearing leg reconstructed by **(B)** CS and **(C)** FBP for 256 spectral projections, **(D)** slice-selective signal intensity maps reconstructed with **(D)** CS and **(E)** FBP, **(F)** NRMSE, **(G)** MAE, and **(H)** SSIM. The FOV of the volume-rendered images **(B**, **C)** is 37.5 mm × 37.5 mm × 37.5 mm. The matrix size of the 3D images is 96 × 96 × 96. The *white* scale bar on the images corresponds to 5 mm. The position of the slice maps is shown in the 3D images with *dashed lines* **(B**, **C)**. Color images are available online.

A mouse tumor-bearing leg (Fig. 4A) was scanned; and CS- and FBP-based 3D EPR signal intensity maps (Fig. 4B, C) were then reconstructed with 256 spectral projections. A threshold of 15% of the maximum signal intensity was applied to the images (Fig. 4B, C) to suppress background noise. The FOV of these 3D images is 37.5 mm × 37.5 mm × 37.5 mm. The matrix size of the 3D images is 96 × 96 × 96. The shape of the tumor-bearing leg and the tail can be seen. However, the FBP-based image (Fig. 4C) has visible background noise above the threshold value. EPR signals were not visualized in some parts of the tumor-bearing legs, such as inside the tumor-bearing leg (Supplementary Video S1). This might be related to inhomogeneous vasculature, tension of the leg that was slightly stretched during the image scan, and the limited sensitivity of EPR detection.

In contrast to the FBP-based image, background noise was suppressed in the CS-based image (Fig. 4B). Slice-selective EPR signal intensity maps (Fig. 4D, E) were obtained from the 3D images reconstructed with CS and FBP. The number of spectral projections used for image reconstruction is indicated above the images. Dashed lines in the 3D images (Fig. 4B, C) show the slice-selective map position. The quality of each 3D image was assessed by NRMSE, MAE, and SSIM (Fig. 4F–H). The CS-based 3D signal intensity map with 256 spectral projections was used as a reference image for NRMSE, MAE, and SSIM for both CS- and FBP-based images. This is because the FBP-based image with 256 spectral projections (Fig. 4C) had significant noise in the background, and consequently, it was not appropriate as a reference image.

### Redox-sensitive mapping of mouse tumor-bearing legs with CS reconstruction

**FIG. 5.**
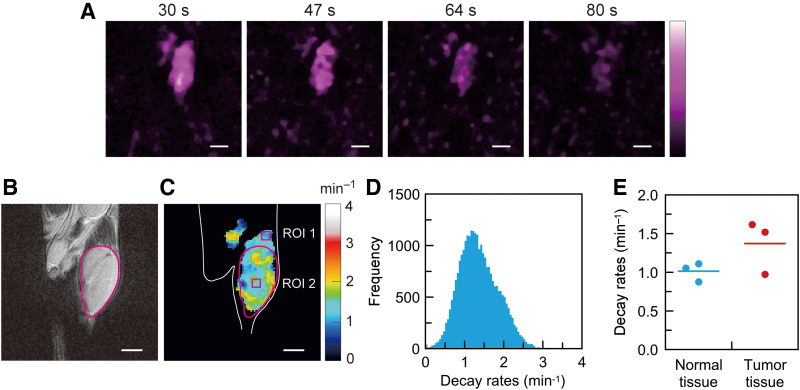
**Redox-sensitive mapping of a mouse tumor-bearing leg using the ^15^N-PDT probe. (A)** EPR signal intensity maps reconstructed by CS (number of projections 128), **(B)** ^1^H-MR anatomical image, **(C)** the decay-rate map of ^15^N-PDT, **(D)** histogram of the decay rates, and **(E)** comparison of the mean decay rates in the ROI 1 (5 × 5 pixels) in normal tissue and ROI 2 (5 × 5 pixels) in tumor tissue **(C)** for three mouse tumor models. The bars in **(E)** represent the mean values for each group. The FOV and the image matrix of the MR image **(B)** are 37.5 mm × 37.5 mm and 120 × 120, respectively. The *white* scale bar corresponds to 5 mm. The tumor outline was obtained from the MR image (*red line*). The outline of the mouse body and leg was drawn by hand on the MR image, and then copied to the decay-rate map **(C)**. The decay-rate map is visually aligned to the edge of the mouse body and leg. MR, magnetic resonance; ROI, region of interest. Color images are available online.

MIA PaCa-2 mouse tumor-bearing legs were measured to demonstrate redox-sensitive mapping based on CS image reconstruction and the sparse spectral sampling strategy. Serial signal intensity maps of ^15^N-PDT in a tumor-bearing leg (Fig. 5A) were acquired starting from 30 s after intravenous injection. While we acquired 12 3D signal intensity maps for the tumor-bearing leg over 201 s, five early 3D maps were used to compute the decay rates voxel by voxel. Later 3D maps had a lower SNR and were not used. The magnetic resonance (MR) anatomical map of the tumor-bearing leg in the coronal plane (Fig. 5B) was scanned as a reference map. This MR anatomical map corresponds to the slice position of the EPR signal intensity maps (Fig. 5A). The decay-rate map (Fig. 5C and Supplementary Video S2) was computed from five signal intensity maps. A threshold of 25% of the maximum signal intensity was applied to the decay-rate computation to avoid background noise in the signal intensity maps. A histogram of the decay rates (Fig. 5D) was obtained from the 3D decay-rate data. The median of the decay rates was 1.31 min^−1^ for the histogram (Fig. 5D). Three mouse tumor models were measured for the decay rates to examine the reproducibility of the present mapping method. The two other histograms of the decay rates are given in the Supplementary Data (Supplementary Fig. S5); the median values were 1.31 and 0.96 min^−1^. To show the alteration of the decay rates in tumor tissue, square region-of-interest (ROI) 1 and 2 were set in normal and tumor tissues (the size of both ROIs was 5 × 5 pixels) (Fig. 5C). We compared the mean decay rates in ROI 1 and 2 for three mouse tumor models (Fig. 5E). The bars in the plots represent the mean values of the decay rates for each group (1.00 and 1.36 min^−1^).

To compare the decay-rate maps based on the FBP and CS approaches, we also obtained a decay-rate map by FBP. FBP-based signal intensity maps, the corresponding decay-rate map, and the histogram of the decay rates were processed from the same spectral data (Supplementary Fig. S6). The FBP-based images had more noise in those backgrounds than the CS-based images shown in Figure 5. FBP-based signal intensity maps with lower SNRs led to the decay-rate map with significant distortion and greater variance as a result. The quantitative comparison of the decay-rate maps is also given in the Supplementary Data (Supplementary Table S1).

## Discussion

### EPR imaging of the solution phantom

Our data acquisition and CS image reconstruction for 3D EPR imaging worked appropriately. The advantages of CS image reconstruction are supported by the results for the solution phantom (Fig. 3). In particular, CS-based images have less background noise and artifacts compared with FBP-based images, even though they require fewer spectral projections. In contrast to CS image reconstruction, “star artifacts” (commonly known as background artifacts for FBP) appeared in the FBP-based images with 256 or fewer spectral projections (Fig. 3B). When we compared the two image-reconstruction approaches, the CS approach is clearly beneficial for background noise suppression with fewer spectral projections. For NRMSE and MAE, these values for CS-based images are always less than the corresponding values for FBP-based images. These results mean that CS image reconstruction could suppress the background noise and artifacts even under a limited number of spectral projections. This advantage is due to L_1_-regularization and a soft-thresholding operator in CS image reconstruction (22). L_1_-regularization is a mathematical technique used in the optimization problem to find a sparse EPR signal distribution. This regularization uses L_1_-norm as a penalty term in the optimization problem. L_1_-norm gives the sum of the absolute values of the elements in the signal distribution array and the sparsity of the signal distribution. The formula for the optimization problem to obtain the signal distribution is given in the section “Materials and Methods.” The SSIM value of the CS-based images with 256 projections almost matches that of the FBP-based image with 1024 projections. From these results, we concluded that fourfold acceleration was achieved for the multiple pillar phantom experiment.

The signal intensity inhomogeneity, which is visible in Figure 2, is due to the resonator's radiofrequency (RF) magnetic field inhomogeneity. In brief, since the EPR signal intensity is proportional to the square of the RF magnetic field, the solutions in the pillars close to higher RF currents in the resonator's coils had higher signal intensities than other solution pillars (16). However, this sensitivity inhomogeneity does not affect the decay-rate mapping in the mouse tumor models because the decay rates are independent of the signal sensitivity distribution.

Johnson *et al.* demonstrated eightfold acceleration of EPR imaging for an isolated rat heart (22); however, available acceleration might depend on the SNR of the measured spectral projections. The measured spatial resolution (2.5 mm) of the images (Fig. 2) was lower than the estimated spatial resolution (0.073 mT/40 mT m^−1^ = 1.8 mm) from the peak-to-peak linewidth of the first-derivative absorption peaks (0.073 mT) and the applied magnetic field gradient (40 mT/m). A large number of spectral projections (16,384 projections) and a state-of-the-art algebraic reconstruction technique recently demonstrated improved spatial resolution (29). While many projections can help improve the spatial resolution, rapidly decaying signals, such as those of ^15^N-PDT or other six-membered nitroxyl radicals, do not allow enough time to obtain spectral projections from the subject mouse. The improvement of the SNR for the measured spectra is also essential to enhance the spatial resolution of EPR images. The resonator and the receiver systems in the EPR spectrometer are critically essential to reduce the noise on the spectral baseline and improve the sensitivity of signal detection. However, such technical investigations on the EPR spectrometer setup are beyond the scope of this article (14, 37, 38, 55).

### Comparison of CS- and FBP-based images for a mouse tumor-bearing leg

The advantage of CS image reconstruction and sparse projection sampling is further shown in EPR imaging of a mouse tumor-bearing leg and rapidly decaying ^15^N-PDT radical. As discussed above, the background noise suppression in the CS approach is clear evidence (Fig. 4B, C). More importantly, the spatial distribution of the EPR signal in the tumor is less sensitive to a decrease in the number of spectral projections. For FBP-based images (Fig. 4E), the EPR signal intensity maps of the tumor-bearing leg were degraded when the number of spectral projections was 64 or 128. In contrast to FBP-based images, the outlines of these signal distributions (64 and 128 projections) remained similar to those in the image with 256 spectral projections in CS-based images (Fig. 4D).

The CS-based image of 256 projections was used as a reference for three quantitative quality assessments for both CS- and FBP-based images. This reference setting is less fair to FBP-based images than to CS-based images. However, since we could not use the noise-free reference image for the mouse tumor-bearing leg, the image with the least noise in the CS approach was used as the reference. Degradation of image quality with fewer projections could be seen (Fig. 4F–H). The CS-based image with 128 spectral projections was better than the FBP-based image with 256 spectral projections in terms of all three indicators and by visual inspection. The total acquisition time for 256 spectra under a magnetic field gradient and the zero-gradient spectrum was 33.4 s because the extra time to back the magnetic field took 30 ms and the duration of field scanning for the spectral acquisition was 100 ms (total 130 ms). Considering the EPR signal lifetime of 1.4 min in the preliminary result, we expected that the acquisition of 256 spectral projections was possible without significant signal decay during spectral acquisition. We finally decided to scan the series of ^15^N-PDT signal intensity maps from the tumor-bearing leg with 128 spectral projections.

### Redox-sensitive mapping of a mouse tumor-bearing leg using ^15^N-PDT

Three-dimensional decay-rate mapping of the xenograft tumors (Fig. 5C) was performed using EPR imaging and the short-lifetime nitroxyl radical ^15^N-PDT for the first time. This has not been reported previously since the acquisition time for 3D CW-EPR imaging was not suited for the signal decay of ^15^N-PDT or other short-lifetime nitroxyl radicals. No statistical test was performed on the data shown in Figure 5E because of the limited sample size; however, the data of Figure 5 demonstrated that the present mapping method and ^15^N-PDT are applicable for redox status monitoring in the mouse tumor models. State-of-the-art fast-scan CW-EPR imagers can potentially measure 3D decay-rate maps of mouse disease models within a short period (1, 10, 39). Nevertheless, CS image reconstruction and the sparse spectral sampling strategy can further accelerate 3D EPR image acquisition. There have been a few descriptions of 3D EPR imaging in small rodents with 6-membered nitroxyl radicals, such as Tempone and 4-hydroxy-2,2,6,6-tetramethyl-1-piperidynyloxyl (Tempol), to date (20, 42). For CW-EPR, spatial imaging of ^15^N-labeled Tempol-d_17_ radical was demonstrated for a mouse head (42). In that study from 2008, the acquisition time of a single 3D image was 30 s for 46 spectral projections. A subject mouse received Tempol-d_17_ radicals *via* the intraperitoneal route. Using a cutting-edge EPR acquisition system, more spectral projections can be recorded from the tumor within a limited time, even with the short-lifetime radical ^15^N-PDT.

Signal intensity maps with higher SNRs are essential to obtain accurate decay-rate maps for mouse tumor models. Therefore, CS image reconstruction and the sparse spectral sampling strategy are more suitable for rapidly decaying nitroxyl radicals such as ^15^N-PDT than the conventional FBP approach when the acquisition time is limited. The comparison of decay-rate mapping with these two approaches (Fig. 5; Supplementary Fig. S6 and Table S1) illustrated the advantage of CS image reconstruction and the sparse sampling strategy. This technological progress in redox-sensitive mapping could be useful when radical probes that are very sensitive to the redox balance are used with a highly reducing environment such as malignant tumors. Remarkable differences in the two approaches are the degradation of the decay-rate map (Supplementary Fig. S6) and an increase in the decay-rate variance (Supplementary Table S1). The acquisition time and the number of spectral projections had always been compromised for EPR-based redox-sensitive mapping using nitroxyl radicals. Nevertheless, the demonstrated acceleration of EPR-based redox-sensitive mapping will contribute to obtaining high-quality redox-sensitive maps in animal studies.

In contrast to CW-EPR, ^15^N-PDT was visualized in the mouse tumor-bearing leg with 300-MHz Pulsed EPR (20). Three-dimensional oxygen mapping was performed using ^15^N-PDT and a single-point imaging modality. This was the first report of probing the intracellular concentrations of oxygen using the cell-permeable nitroxyl radical ^15^N-PDT. Since pulsed EPR requires a spin probe with a longer relaxation time, nitroxyl radicals are generally excluded, except for ^15^N-PDT. However, CW-EPR can measure nitroxyl radicals regardless of the relaxation time. Therefore, CW-EPR is suitable for 3D redox-sensitive mapping using nitroxyl radicals. If we use CS image reconstruction, the sparse sampling strategy, and fast-scan CW-EPR, various nitroxyl radicals with different degrees of lipophilicity and different sensitivities to the redox state can be used for specific applications.

In previous studies, redox mapping of mouse tumor-bearing legs was performed with ^1^H-MRI using signal enhancement due to exogenously injected nitroxyl radicals (19, 34). In the literature (19), Tempol has also been used for redox mapping of tumors. The decay rate of Tempol in squamous cell carcinoma (SCC VII) tumors was reported to be 1.1 ± 0.2 min^−1^. While the nitroxyl radicals and tumors used are different from those in our animal experiments, the decay rates of the six-membered nitroxyl radicals (^15^N-PDT and Tempol) in tumors were comparable. The preliminary measurements in overall tumor-bearing legs might include some normal tissues that had a smaller decay rate (longer lifetime). Also, the threshold processing for the decay-rate computation might reduce the detected volume of normal tissue with a lower signal intensity. These considerations may explain the difference between the median of the mapped decay rates (1.31 min^−1^, Fig. 5D) and the preliminary measured decay rate (0.71 min^−1^). Studies on MRI-based redox mapping using nitroxyl radicals have shown that the time-dependent disappearance of MR image intensity enhancement occurred as “the reduction of nitroxyl radical rather than clearance” (34). Since our experiments with mouse xenograft models and nitroxyl radicals are similar to prior studies on redox mapping using MRI (19, 34), this suggests that the EPR signal intensity decay in tumors predominantly reflected the reduction of ^15^N-PDT rather than clearance in the present work.

An advantage of EPR-based redox-sensitive mapping is the higher sensitivity to nitroxyl radicals. ^1^H-MRI-based redox mapping uses signal enhancement due to nitroxyl radicals. Therefore, ^1^H-MRI indirectly detects the redox state through the contrast enhancement of nitroxyl radicals. Nevertheless, the contrast enhancement in MRI with nitroxyl radicals is weaker than that of clinically used gadolinium contrast agents. Therefore, ^1^H-MRI-based redox mapping required a higher dose of nitroxyl radicals (Tempol, 1.6 μmol/g body weight) (19). This dose is 1.8-fold higher than our ^15^N-PDT dose (0.9 μmol/g body weight) (see “Materials and Methods” section). In contrast, EPR directly detects unpaired electrons and therefore has higher detection sensitivity. However, MRI has good spatial resolution that is superior to EPR imaging with limited spectral projections. Moreover, ^1^H-MRI provides an anatomical map of a subject animal and image contrast for tumor tissues. These features are advantageous in coimaging EPR-based decay rates and MR anatomical information (Fig. 5B, C).

EPR-based redox mapping using nitroxyl radicals has been applied to the brain (45), lung and stomach (49), liver (23), and tumors (30) in model diseases involving oxidative stress such as ischemia-reperfusion, hypoxia, exposure to diesel exhaust particles, and iron overload, and is a powerful tool for evaluation of the severity of these diseases. These studies using redox mapping are based on the decay rate of nitroxyl radicals. They are attributable to the reduction of nitroxyl radicals by reduced thiol, including glutathione (GSH) in tissues (30, 46). GSH is rapidly consumed by ROS in oxidative stress-related diseases and is reflected in redox-sensitive mapping. In tumor tissue, intermittent (cycling) hypoxic regions have been reported, and are known to confer resistance to radiotherapy and chemotherapy (6). It has recently become clear that this intermittent hypoxic region can be altered within several minutes, speculating that spatial redox status is also synchronized with this ischemia-reperfusion cycle in tumors (54). This study reported that the temporal resolution in EPR-based redox-sensitive mapping could be dramatically shortened by using fast decaying nitroxyl radicals. Therefore, visualization of a shift in the redox state that occurred within the order of several minutes may be possible. In the future, this technique will be a useful tool for redox mapping of oxidative stress-related diseases, such as intermittent hypoxia in a tumor, where GSH changes in a short time for a few minutes.

In conclusion, an accelerated EPR-based redox-sensitive mapping system was established. Redox-sensitive mapping in a mouse xenograft model was demonstrated with EPR imaging of nitroxyl radicals and the sparse projection sampling strategy. Due to this progress in EPR-based redox-sensitive mapping, better image quality for a redox-sensitive map could be achieved with a limited number of spectral projections for various applications. This enables the use of various nitroxyl radical probes, including short-lifetime nitroxyl radicals. This advancement can overcome the limitations of the acquisition time of 3D EPR mapping and the selection of nitroxyl radicals that have a short lifetime in living animals.

## Materials and Methods

### Redox-sensitive mapping

The reduction reaction of a nitroxyl radical results in its hydroxylamine form and a loss of EPR absorption (48). Therefore, the decay rate of EPR signals over time reflects the redox state in the environment; that is, the biological tissue in our study. The 3D map of the decay rates of the EPR signal intensity was obtained by exponential curve fitting of the EPR signal time-course voxel by voxel, as illustrated in Figure 1B.

### Sparse projection sampling and image reconstruction

Three-dimensional mapping of the EPR signal intensity was reconstructed from spectral projections in CW-EPR detection under a magnetic field gradient. The magnetic field gradient directions were set by the GM sampling strategy, as shown in Figure 1C, to acquire the spectral projections in 3D space uniformly. The 3D GM sampling method determined the points on a sphere derived from the golden ratio (5). This sampling strategy provided a relatively uniform distribution of projections on a sphere. The projection numbers were set at 64, 128, 256, 512, 1024, and 2048 for a full sphere.

FBP and CS image reconstruction were implemented for EPR spectral projections. For FBP, 3D one-step reconstruction was performed with the projected signal profiles computed from the spectral projections. For CS image reconstruction, we implemented the reconstruction code according to Johnson *et al.* (22). The optimization problem for image *x* was formulated as

(1) $$x_{cs}=\arg\min_{x}{∥CRx-y∥}_{2}^{2}+\lambda_{1}{∥x∥}_{1}^{1}+\lambda_{2}{∥TV\left(x\right)∥}_{1}^{1},$$

where *x*_CS_ is the solution of the optimization problem, *C* is the convolution operator, *R* is the Radon transform, and *y* is the observed spectral projections. TV is the total variation, and *λ*_1_ and *λ*_2_ are the weight coefficients of the regularization terms. The reconstruction algorithm for solving Equation (1) was based on FISTA (2). In image reconstruction, we empirically set coefficients *λ*_1_ and *λ*_2_ to 0.1 and 0.01, respectively, for the solution phantom; 0.01 and 0.01, respectively, for tumor-bearing legs. The SNR of EPR signals in animal experiments was lower than that in the multiple pillar phantom. Moreover, the edge of the EPR signal distribution for the tumor-bearing legs was less steep than that in the multiple pillar phantom. These circumstances required a smaller value of *λ*_1_ to obtain the right image for tumor-bearing legs. When the signal distribution sparsity was strong with an enormous value of *λ*_1_, the signal distribution was oversuppressed and strongly distorted. We chose the coefficients *λ*_1_ and *λ*_2_ to obtain the right image by visual inspection.

Iterative processing was performed to find the solution (the signal intensity map). Processing was terminated after four iterations because we obtained reasonable reconstructed images and convergence. Computation codes were implemented for FBP and CS image reconstruction with MATLAB R2020a (Mathworks, Natick, MA). In addition to individual image reconstruction, another constraint was applied to 3D EPR signal intensity maps to maintain agreement with the temporal change in the total amount of nitroxyl radicals. Since our CS image-reconstruction code included the image intensity normalization process to improve the stability of reconstruction computation, the temporal change in the signal distribution cannot be recovered in the series of images. Therefore, we multiplied the mean value of the double-integrated signal amplitudes of the first-derivative EPR spectral projections in each data set by the corresponding reconstructed images to recover the signal decay profile for all CS-based images.

### Chemicals

The redox-sensitive nitroxyl radical ^15^N-PDT (product no. M-2327) was purchased from CDN Isotopes (Quebec, Canada). All chemicals used were of analytical grade.

### CW-EPR spectrometer/imager

A 750-MHz home-built CW-EPR spectrometer/imager was used for the EPR measurements. The details of the spectrometer/imager have been reported previously (43). In brief, an RF signal generator (E8257D; Keysight Technologies, Santa Rosa, CA) was used as an RF source. A multicoil parallel-gap resonator, which has a sample space of 22 mm diameter and 30 mm long, was used in the reflection-type EPR bridge (26). The permanent magnet system (static magnetic field 27 mT; NEOMAX Corp., a subsidiary of Hitachi Metals, Ltd., Tokyo) was used with the coils of magnetic field gradients and the magnetic field scanning coil. EPR signal recording, magnetic field gradients, and field scanning were controlled with dedicated LabVIEW software. The data acquisition boards (PCIe-6251 and PCIe-6259; National Instruments, Austin, TX) were equipped with an Apple Mac Pro computer.

### Phantom preparation

A cylinder (21.8 mm diameter and 22.5 mm long) made of crosslinked polystyrene (Rexolite^®^ 1422) had 10 holes (3.0 mm diameter and 15.0 mm long) at a spacing of 5.0 mm. This was called the multiple pillar phantom. Each hole was filled with ^15^N-PDT (2 mmol/L) dissolved in phosphate-buffered saline (PBS) (1.06 mL in total). ^15^N-PDT solutions were freshly prepared and air saturated.

### Animal preparation

All animal experiments were performed under the “Law for The Care and Welfare of Animals in Japan” and approved by the Animal Experiment Committee of Hokkaido University (approval no. 20-0118). Six-week-old BALB/c-*nu*/*nu* male mice were purchased from Japan SLC (Hamamatsu, Japan) and housed at five mice per cage in a climate-controlled room with 12-h light 12-h dark cycle and room temperature of 23°C. They had free access to standard mouse diet (Labo MR Stock, Sankyo Labo Service Corp., Inc., Tokyo, Japan) and water *ad libitum*. The mice were acclimated to their environment for a week before cell inoculation. The human-derived pancreatic ductal adenocarcinoma cell line MIA PaCa-2 was obtained from the American Type Culture Collection (ATCC, Manassas, VA). Approximately 10 million cells of MIA PaCa-2 were subcutaneously inoculated into the right hind legs of BALB/c-*nu*/*nu* mice. Tumors were developed over three weeks until the tumor volume reached ∼900–1000 mm^3^. The mice were 10–11 weeks old, and weighed 23–25 g at the time of EPR and MR image scanning. We did not use an electronic laboratory notebook for our study.

### EPR imaging of the solution phantom

The multiple pillar phantom was placed in the resonator of the EPR imager. The measurement parameters for EPR imaging were as follows: incident RF power 2.8 mW, magnetic field scanning 1.5 mT, field scan duration 100 ms, magnetic field modulation 0.06 mT, modulation frequency 90 kHz, time constant of lock-in amplifier 0.1 ms, number of data acquisition 512 points per scan, and magnetic field gradient 40 mT/m. The central magnetic field was set at a lower field of two EPR absorption peaks of ^15^N-PDT. The conversion efficiency of the RF magnetic field was 126 μT/W^1/2^. The direction of the magnetic field gradient was controlled with the GM strategy, as described above. The projection numbers were set to 2048. The single-image acquisition time was 266 s. EPR spectroscopy and imaging of the phantom were conducted at room temperature. The spin system was below saturation and in the linear response region.

### EPR imaging of a mouse tumor-bearing leg

Mouse tumor xenograft models were measured to demonstrate redox-sensitive mapping with sparse sampling of EPR spectral projections. For redox-sensitive mapping, a tumor-bearing mouse was anesthetized by inhalation of isoflurane (initially 2.5% and then 1.5%–2.0%). The subject mouse was placed in a mouse holder in a prone position. The tumor-bearing leg of the subject mouse was placed in the RF resonator. The tail vein was cannulated for the intravenous injection of nitroxyl radical. Mouse body temperature was monitored from the rectum with a fiber optic temperature sensor, and the respiration of the mouse was also monitored with a small air-pressure sensor. The body temperature was maintained at 37°C by feedback-regulated heated airflow with an MR-compatible small animal monitor and air heater system (Model 1025; SA Instruments, Inc., Stony Brook, NY). The respiration rate was maintained at 50 ± 10 breaths per minute.

The ^15^N-PDT probe (0.9 μmol/g body weight) dissolved in PBS (pH 7.4) was intravenously injected into a subject mouse *via* the tail vein over 30 s. This dose amount in EPR imaging of mouse tumor models was determined to give better SNRs of the EPR signals. Thirty seconds after the injection, EPR imaging acquisition was started. The measurement parameters of *in vivo* EPR imaging were as follows: incident RF power 9.1 mW, magnetic field scanning 1.5 mT, field scan duration 100 ms, magnetic field modulation 0.06 mT, time constant of lock-in amplifier 0.1 ms, number of data acquisition 512 points per scan, and maximum field gradient 30 mT/m. The total acquisition time was 33.4 s for a single imaging scan for 256 spectral projections and the zero-gradient spectrum to test our CS reconstruction code. Sequential data acquisition was also performed to record the time course of the signal intensity in tumors for redox-sensitive mapping by repeating the acquisition of 128 spectral projections and the zero-gradient spectrum (16.8 s).

### ^1^H-MRI of a mouse tumor-bearing leg

Immediately after ERP imaging, MRI measurements were performed on a home-built 1.5 T permanent magnet system, using a ^1^H mouse body coil (30 mm inner diameter; Takashima Seisakusho Co., Ltd., Tokyo, Japan) and a dedicated spectrometer (Japan REDOX, Ltd., Fukuoka, Japan). The same mouse bed was used for both EPR and MRI to ensure approximately equivalent scan positioning. T_2_-weighted anatomical two-dimensional images of the tumor-bearing legs on a coronal plane were obtained using a fast spin echo sequence with the following parameters: FOV 80 mm × 80 mm, 256 × 256 in-plane matrix, eight slices of 2 mm thickness, echo time (TE)/repetition time 16/3000 ms, effective TE 64 ms, echo train 4, number of averages 4, and acquisition time 13 min.

### Image-quality assessment

For the multiple pillar phantom and the mouse tumor-bearing legs, the reconstructed images were assessed in terms of three indicators; that is, NRMSE, MAE, and SSIM (51). These indicators are given as

(2) $$NRMSE=\frac{1}{x_{ref\max}-x_{ref\min}}\sqrt{\frac{1}{N}{\sum_{i=1}^{N}\left[x_{ref}\left(i\right)-x_{recon}\left(i\right)\right]}^{2}},$$

(3) $$MAE=\frac{1}{N}\sum_{i=1}^{N}\left|\frac{x_{ref}\left(i\right)}{x_{ref\max}}-\frac{x_{recon}\left(i\right)}{x_{recon\max}}\right|,$$

(4) $$SSIM=\frac{\left(2\mu_{ref}\mu_{recon}+c_{1}\right)\left(2\sigma_{refrecon}+c_{2}\right)}{\left(\mu_{{}_{ref}}^{2}+\mu_{{}_{recon}}^{2}+c_{1}\right)\left(\sigma_{ref}^{2}+\sigma_{recon}^{2}+c_{2}\right)},$$

where *x*_ref_ and *x*_recon_ are the reference image and the reconstructed image, respectively. Moreover, *x*_ref max_ and *x*_ref min_ are the maximum intensity and the minimum intensity of *x*_ref_, respectively. *N* is the total number of voxels being evaluated. Notations μ_ref_ and μ_recon_ are the averages of *x*_ref_ and *x*_recon_, respectively. Also, σ_ref_^2^ and σ_recon_^2^ are the variances of *x*_ref_ and *x*_recon_, respectively, and σ_ref recon_ is the covariance of *x*_ref_ and *x*_recon_. The variables *c*_1_ and *c*_2_ are used to stabilize the division with a weak denominator. Function “ssim” in MATLAB was used to obtain the SSIM of the reconstructed images. The variables *c*_1_ and *c*_2_ were given as default values in function “ssim.” NRMSE and MAE were obtained by our MATLAB codes.

## Supplementary Material

Supplemental data

Supplemental data

Supplemental data

Supplemental data

Supplemental data

Supplemental data

Supplemental data

Supplemental data

Supplemental data

Supplemental data

## Abbreviations Used

^15^N-PDT: 4-oxo-2,2,6,6-tetramethylpiperidine-d_16_;1-^15^N-1-oxyl
1D: one dimensional
3D: three dimensional
4-oxo-TEMPO: 4-oxo-2,2,6,6-tetramethylpiperidine-N-oxyl
CS: compressed sensing
CW: continuous wave
DNP: dynamic nuclear polarization
EPR: electron paramagnetic resonance
FBP: filtered back-projection
FISTA: fast iterative shrinkage-thresholding algorithm
FOV: field of view
FWHM: full-width at half-maximum
GM: golden means
GSH: glutathione
MAE: mean absolute error
MR: magnetic resonance
MRI: magnetic resonance imaging
NRMSE: normalized root mean square error
PBS: phosphate-buffered saline
RF: radiofrequency
ROI: region of interest
ROS: reactive oxygen species
SNR: signal-to-noise ratio
SSIM: structural similarity
Tempol: 4-hydroxy-2,2,6,6-tetramethyl-1-piperidynyloxyl
TV: total variation
