# Supplementary material for: Redox-Sensitive Mapping of a Mouse Tumor Model Using Sparse Projection Sampling of Electron Paramagnetic Resonance
Source: Antioxid Redox Signal. 2022 Jan 17;36(1-3):57–69. doi: 10.1089/ars.2021.0003 (PMC8823265; doi:10.1089/ars.2021.0003)
Supplement: Supplemental data [file Supp_DataS1.pdf]

Supplementary Information

Redox-sensitive mapping of a mouse tumor model using sparse projection sampling of electron paramagnetic resonance

Kota Kimura,[1] Nami Iguchi,[1] Hitomi Nakano,[2] Hironobu Yasui,[3] Shingo Matsumoto,[2] Osamu Inanami,[3] Hiroshi Hirata[2,*]

[1] Division of Bioengineering and Bioinformatics, Graduate School of Information Science and Technology, Hokkaido University, North 14, West 9, Kita-ku, Sapporo, 060-0814, Japan

[2] Division of Bioengineering and Bioinformatics, Faculty of Information Science and Technology, Hokkaido University, North 14, West 9, Kita-ku, Sapporo, 060-0814, Japan

[3] Laboratory of Radiation Biology, Faculty of Veterinary Medicine, Hokkaido University, North 18, West 9, Kita-ku, Sapporo, 060-0818, Japan

* Hiroshi Hirata, Ph.D.
E-mail: hhirata@ist.hokudai.ac.jp

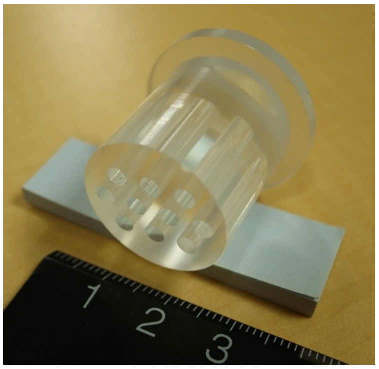

Figure S1. Photograph of the multiple pillar phantom. The reference scale in the photo is in centimeters.

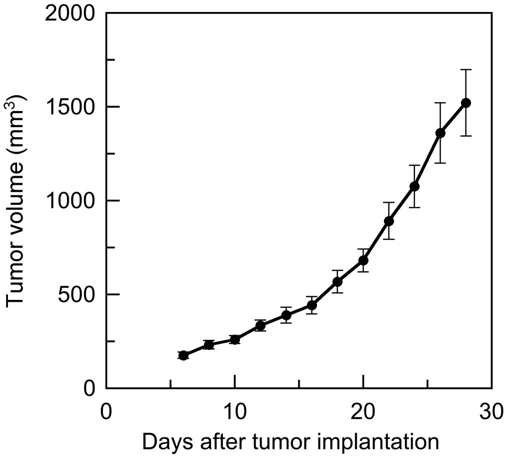

Figure S2. Tumor growth curve of mouse xenograft models of the human-derived pancreatic ductal adenocarcinoma cell line MIA PaCa-2. The plots and error bars show the mean and standard error of the mean (SEM) of tumor volumes (sample size $n = 8$). The tumor volume (V) was measured with linear calipers and calculated as V = (length × width × depth) π/6. Note that 7 of 8 mice received nitroxyl radicals for measuring the time-course of EPR signal intensity when the tumor volume reached approximately 900 to 1000 mm^3.

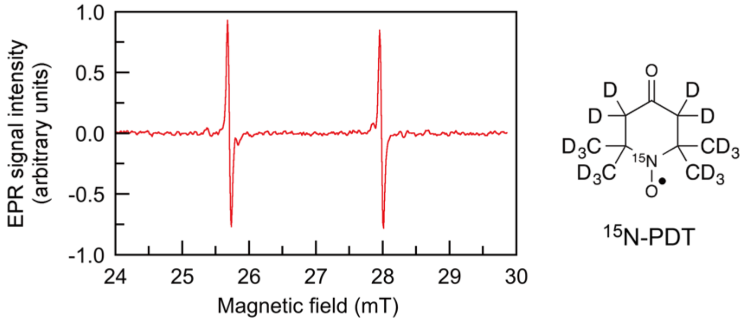

Figure S3. Representative first-derivative EPR spectrum for ^{15}N-PDT measured from a mouse tumor-bearing leg. This spectrum was obtained at the peak of the time-course of EPR signal intensity.

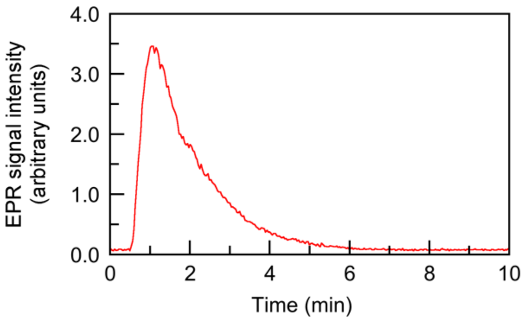

Figure S4. Representative time-course of EPR signals for ^{15}N-PDT (0.4 μmol/g body weight) intravenously injected into a mouse xenograft model (MIA PaCa-2). The tumor-bearing leg was placed in the resonator and the EPR signal from the tumor-bearing leg was recorded continuously. The intravenous injection was started at 0.5 min and finished at 1.0 min in the time axis. The EPR signal quickly appeared and decayed after the peak. In our preliminary results, the mean lifetime of exponential decay for ^{15}N-PDT EPR signal was estimated to be 1.4 min based on three individual measurements. This lifetime corresponds to a half-life of 1.0 min and a decay rate of 0.71 min^{-1}. For the mouse xenograft model, which was used to obtain the data (Fig. S4), the body weight was 23.4 g and the tumor volume was 1010 mm^3.

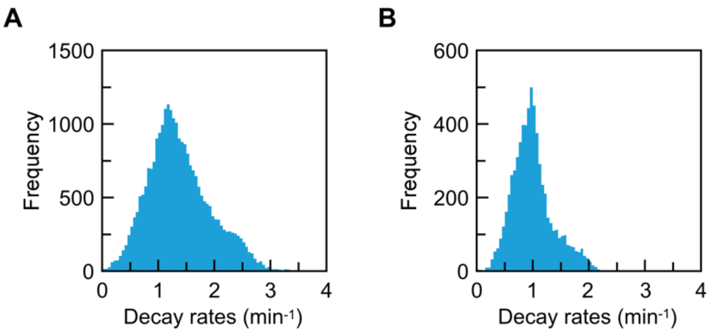

Figure S5. Histograms of the decay rates of ^{15}N-PDT in the mouse tumor-bearing legs for (A) mouse #2 and (B) mouse #3. The histogram of the decay rates for mouse #1 is given in the main text (Fig. 5D). A threshold of 25% maximum signal intensity was applied to the decay-rate computation. The medians of the decay rates are 1.31 min^{-1} (A) and 0.96 min^{-1} (B).

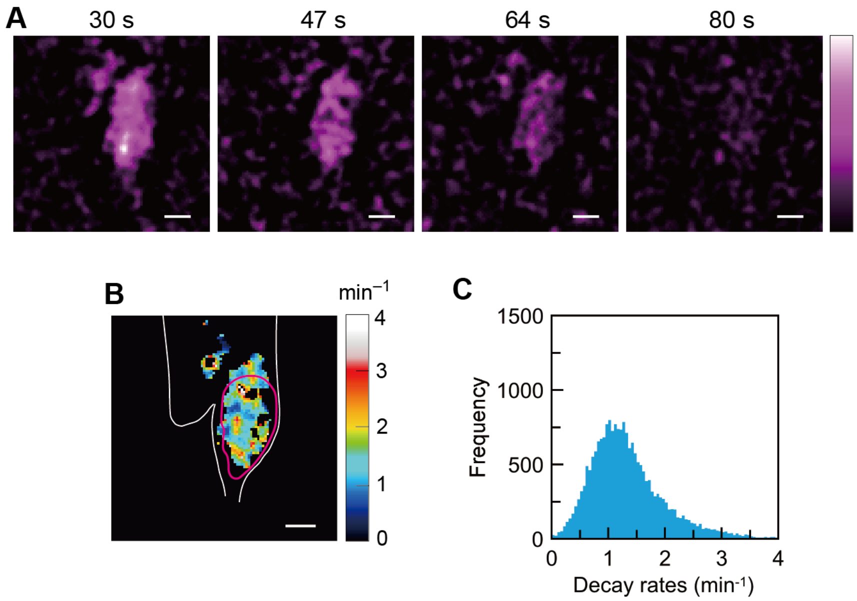

Figure S6. FBP-based redox-sensitive mapping of a mouse tumor-bearing leg using the ^{15}N-PDT probe. (A) EPR signal intensity maps reconstructed by FBP (number of projections 128, the same data for the maps in Fig. 5), (B) the decay-rate map of ^{15}N-PDT, (C) histogram of the decay rates. The white scale bar corresponds to 5 mm. The tumor outline was obtained from the MR image (red line, Fig. 5B). The mouse body and leg outlines were drawn by hand on the MR image (Fig. 5B) and then copied to the decay-rate map (Fig. S6B).

Table S1. Quantitative comparison of the decay rates obtained by FBP and CS approaches.

Image reconstruction	Compressed sensing (CS) (Fig. 5)	Filtered back-projection (FBP) (Fig. S6)
Total voxel counts	27,332	20,495
Mean	1.36 min^{-1}	1.35 min^{-1}
Median	1.31 min^{-1}	1.23 min^{-1}
Standard deviation	0.50 min^{-1}	0.70 min^{-1}
