# Supplementary material for: Redox-Sensitive Mapping of a Mouse Tumor Model Using Sparse Projection Sampling of Electron Paramagnetic Resonance
Source: Antioxid Redox Signal. 2022 Jan 17;36(1-3):57–69. doi: 10.1089/ars.2021.0003 (PMC8823265; doi:10.1089/ars.2021.0003)
Supplement: Supplemental data [file Supp_FigureS2.pdf]

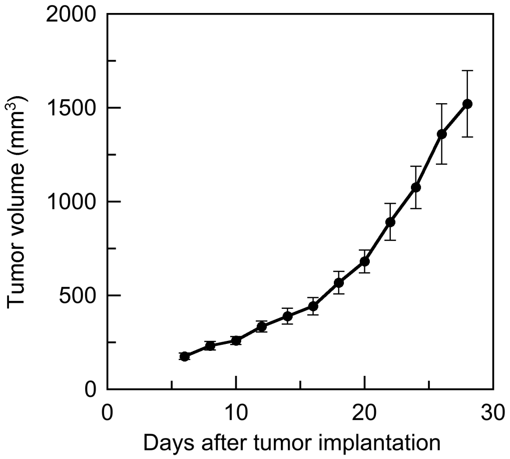

Figure S2. Tumor growth curve of mouse xenograft models of the human-derived pancreatic ductal adenocarcinoma cell line MIA PaCa-2. The plots and error bars show the mean and standard error of the mean (SEM) of tumor volumes (sample size $n = 8$). The tumor volume (V) was measured with linear calipers and calculated as $V = (\text{length} \times \text{width} \times \text{depth})\ \pi/6$. Note that 7 of 8 mice received nitroxyl radicals for measuring the time-course of EPR signal intensity when the tumor volume reached approximately 900 to 1000 mm^3.
