# Supplementary material for: Redox-Sensitive Mapping of a Mouse Tumor Model Using Sparse Projection Sampling of Electron Paramagnetic Resonance
Source: Antioxid Redox Signal. 2022 Jan 17;36(1-3):57–69. doi: 10.1089/ars.2021.0003 (PMC8823265; doi:10.1089/ars.2021.0003)
Supplement: Supplemental data [file Supp_FigureS3.pdf]

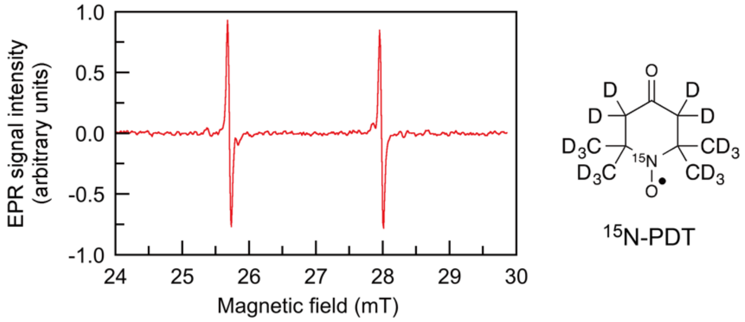

Figure S3. Representative first-derivative EPR spectrum for ^{15}N-PDT measured from a mouse tumor-bearing leg. This spectrum was obtained at the peak of the time-course of EPR signal intensity.
