# Supplementary material for: Redox-Sensitive Mapping of a Mouse Tumor Model Using Sparse Projection Sampling of Electron Paramagnetic Resonance
Source: Antioxid Redox Signal. 2022 Jan 17;36(1-3):57–69. doi: 10.1089/ars.2021.0003 (PMC8823265; doi:10.1089/ars.2021.0003)
Supplement: Supplemental data [file Supp_FigureS4.pdf]

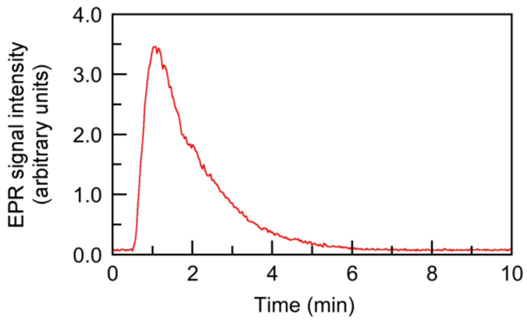

Figure S4. Representative time-course of EPR signals for ^{15}N-PDT (0.4 µmol/g body weight) intravenously injected into a mouse xenograft model (MIA PaCa-2). The tumor-bearing leg was placed in the resonator and the EPR signal from the tumor-bearing leg was recorded continuously. The intravenous injection was started at 0.5 min and finished at 1.0 min in the time axis. The EPR signal quickly appeared and decayed after the peak. In our preliminary results, the mean lifetime of exponential decay for ^{15}N-PDT EPR signal was estimated to be 1.4 min based on three individual measurements. This lifetime corresponds to a half-life of 1.0 min and a decay rate of 0.71 min^{-1}. For the mouse xenograft model, which was used to obtain the data (Fig. S4), the body weight was 23.4 g and the tumor volume was 1010 mm^3.
