# Supplementary material for: Redox-Sensitive Mapping of a Mouse Tumor Model Using Sparse Projection Sampling of Electron Paramagnetic Resonance
Source: Antioxid Redox Signal. 2022 Jan 17;36(1-3):57–69. doi: 10.1089/ars.2021.0003 (PMC8823265; doi:10.1089/ars.2021.0003)
Supplement: Supplemental data [file Supp_VideoS1.zip › KimuraSupplVideoS1.pptx]

## Slide 1
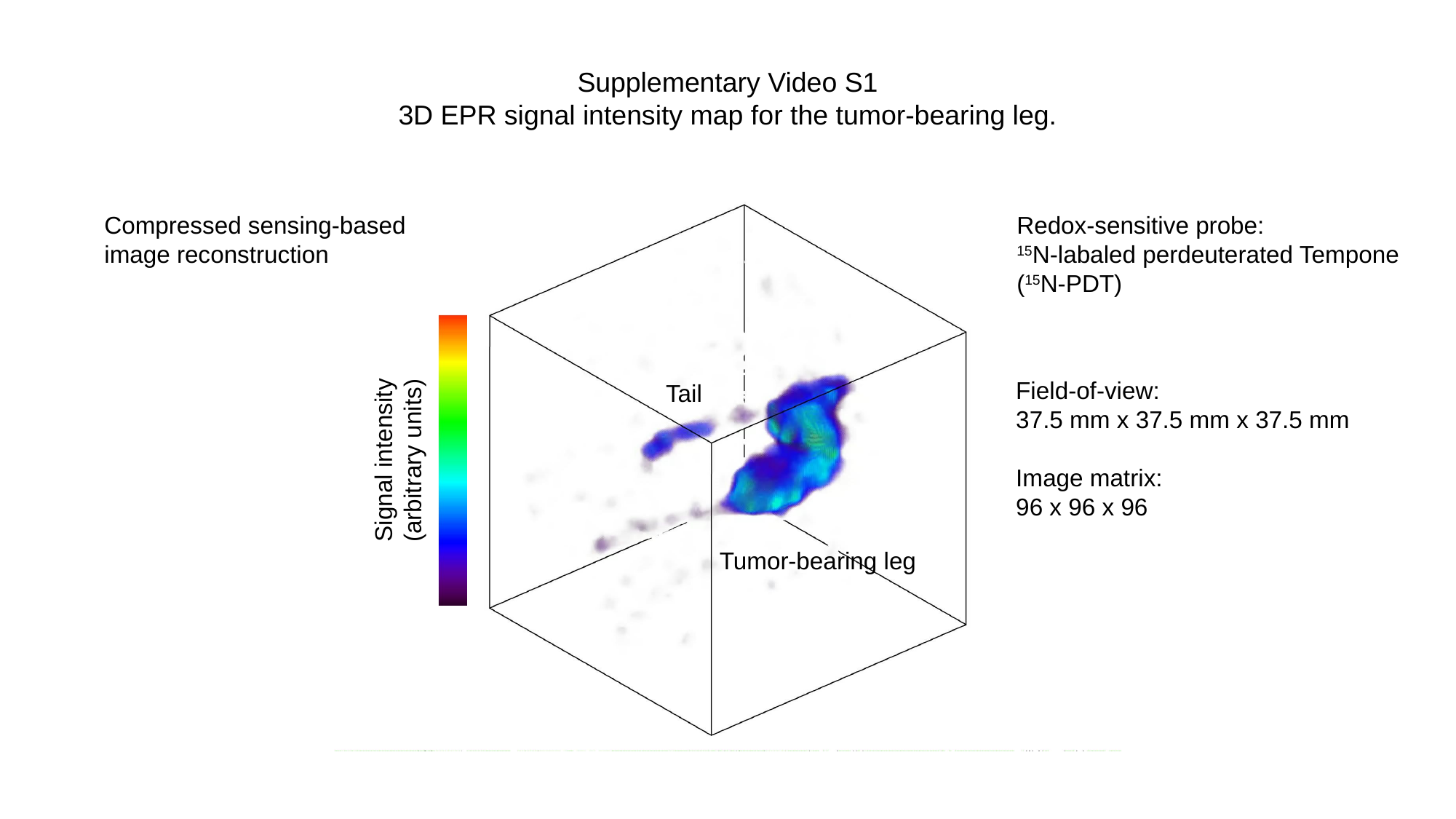

Supplementary Video S1
3D EPR signal intensity map for the tumor-bearing leg.
Compressed sensing-based
image reconstruction
Redox-sensitive probe:
15N-labaled perdeuterated Tempone
(15N-PDT)
Field-of-view:
37.5 mm x 37.5 mm x 37.5 mm
Image matrix:
96 x 96 x 96
Tail
Signal intensity
(arbitrary units)
Tumor-bearing leg
