# Supplementary material for: Redox-Sensitive Mapping of a Mouse Tumor Model Using Sparse Projection Sampling of Electron Paramagnetic Resonance
Source: Antioxid Redox Signal. 2022 Jan 17;36(1-3):57–69. doi: 10.1089/ars.2021.0003 (PMC8823265; doi:10.1089/ars.2021.0003)
Supplement: Supplemental data [file Supp_VideoS2.zip › KimuraSupplVideoS2.pptx]

## Slide 1
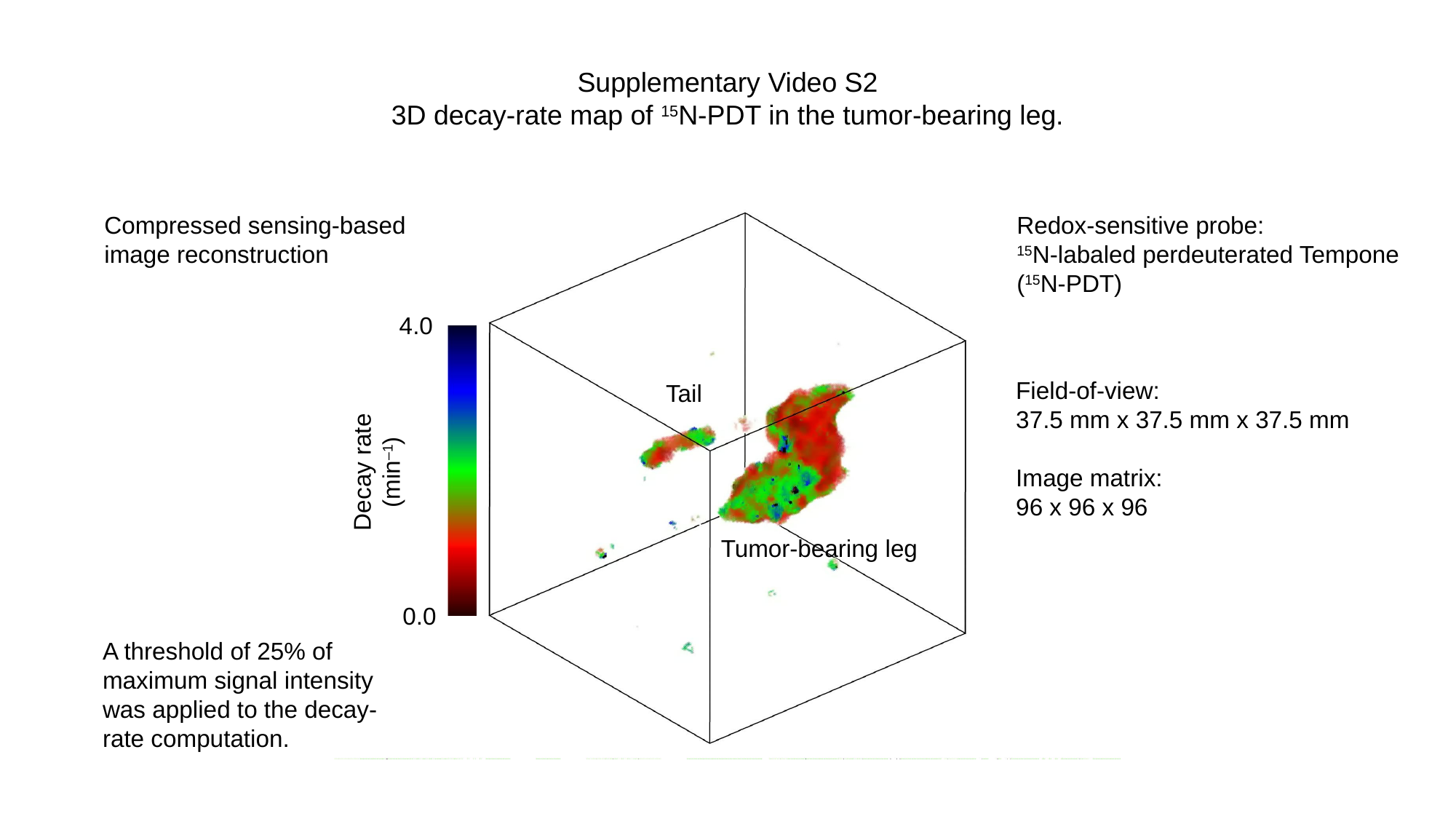

Supplementary Video S2
3D decay-rate map of 15N-PDT in the tumor-bearing leg.
Compressed sensing-based
image reconstruction
Redox-sensitive probe:
15N-labaled perdeuterated Tempone
(15N-PDT)
4.0
Field-of-view:
37.5 mm x 37.5 mm x 37.5 mm
Image matrix:
96 x 96 x 96
Tail
Decay rate
(min–1)
Tumor-bearing leg
0.0
A threshold of 25% of maximum signal intensity was applied to the decay-rate computation.
