# Supplementary material for: Redox-Sensitive Mapping of a Mouse Tumor Model Using Sparse Projection Sampling of Electron Paramagnetic Resonance
Source: Antioxid Redox Signal. 2022 Jan 17;36(1-3):57–69. doi: 10.1089/ars.2021.0003 (PMC8823265; doi:10.1089/ars.2021.0003)
Supplement: Supplemental data [file Supp_FigureS5.pdf]

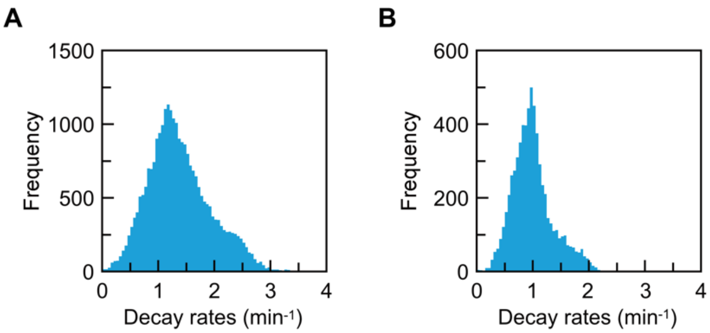

Figure S5. Histograms of the decay rates of ^{15}N-PDT in the mouse tumor-bearing legs for (A) mouse #2 and (B) mouse #3. The histogram of the decay rates for mouse #1 is given in the main text (Fig. 5D). A threshold of 25% maximum signal intensity was applied to the decay-rate computation. The medians of the decay rates are 1.31 min^{-1} (A) and 0.96 min^{-1} (B).
