# Supplementary material for: Redox-Sensitive Mapping of a Mouse Tumor Model Using Sparse Projection Sampling of Electron Paramagnetic Resonance
Source: Antioxid Redox Signal. 2022 Jan 17;36(1-3):57–69. doi: 10.1089/ars.2021.0003 (PMC8823265; doi:10.1089/ars.2021.0003)
Supplement: Supplemental data [file Supp_FigureS6.pdf]

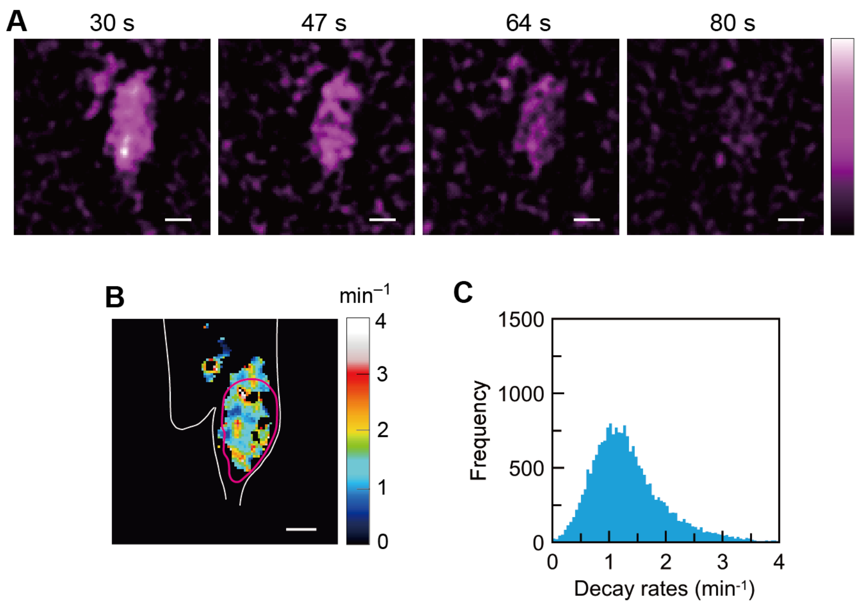

Figure S6. FBP-based redox-sensitive mapping of a mouse tumor-bearing leg using the ^{15}N-PDT probe. (A) EPR signal intensity maps reconstructed by FBP (number of projections 128, the same data for the maps in Fig. 5), (B) the decay-rate map of ^{15}N-PDT, (C) histogram of the decay rates. The white scale bar corresponds to 5 mm. The tumor outline was obtained from the MR image (red line, Fig. 5B). The mouse body and leg outlines were drawn by hand on the MR image (Fig. 5B) and then copied to the decay-rate map (Fig. S6B).
