# Supplementary material for: Redox-Sensitive Mapping of a Mouse Tumor Model Using Sparse Projection Sampling of Electron Paramagnetic Resonance
Source: Antioxid Redox Signal. 2022 Jan 17;36(1-3):57–69. doi: 10.1089/ars.2021.0003 (PMC8823265; doi:10.1089/ars.2021.0003)
Supplement: Supplemental data [file Supp_TableS1.pdf]

Table S1. Quantitative comparison of the decay rates obtained by FBP and CS approaches.

Image reconstruction	Compressed sensing (CS) (Fig. 5)	Filtered back-projection (FBP) (Fig. S6)
Total voxel counts	27,332	20,495
Mean	1.36 min^{-1}	1.35 min^{-1}
Median	1.31 min^{-1}	1.23 min^{-1}
Standard deviation	0.50 min^{-1}	0.70 min^{-1}
